# Circulating PCSK9 levels and CETP plasma activity are independently associated in patients with metabolic diseases

**DOI:** 10.1186/s12933-016-0428-z

**Published:** 2016-08-04

**Authors:** Josefa Girona, Daiana Ibarretxe, Nuria Plana, Sandra Guaita-Esteruelas, Nuria Amigo, Mercedes Heras, Luis Masana

**Affiliations:** 1 Vascular Medicine and Metabolism Unit, Research Unit on Lipids and Atherosclerosis, Sant Joan University Hospital, Universitat Rovira i Virgili, IISPV, C Sant Llorenç, 21, 43201 Reus, Spain; 2 Biosfer Teslab, Reus and Department of Electronic Engineering, Universitat Rovira i Virgili, IISPV, Tarragona, Spain; 3 Spanish Biomedical Research Centre in Diabetes and AssociatedMetabolic Disorders (CIBERDEM), Madrid, Spain

**Keywords:** PCSK9, CETP, Lipids, rs11591147, Metabolic syndrome

## Abstract

**Background:**

PCSK9 inhibition is a new powerful cholesterol-lowering strategy. Recently, it was reported that CETP inhibitors influence PCSK9 levels as an off-target effect. We explored the relationship between circulating PCSK9 levels and CETP activity in patients with metabolic disease who were not on lipid-lowering therapy.

**Methods:**

Plasma CETP activity and PCSK9 levels were measured in 450 participants (median age, 58 years; 49 % women) who attended the metabolism unit because of metabolic syndrome (MetS) (78 %), atherogenic dyslipidemia (32 %), obesity (50 %), type 2 diabetes mellitus (72 %), and other risk factors (13 %). A 6 week lipid-lowering drug wash-out period was established in treated patients.

**Results:**

Both PCSK9 levels and CETP activity were higher in patients with an increasing number of MetS components. PCSK9 levels were positively correlated with CETP activity in the entire cohort (r = 0.256, *P* < 0.0001) independent of age, gender, body mass index (BMI), systolic blood pressure (SBP), LDL cholesterol (LDL-C), triglycerides and glucose. Individuals with the loss-of-function PCSK9 genetic variant rs11591147 (R46L) had lower levels of PCSK9 (36.5 %, *P* < 0.0001) and LDL-C (17.8 %, *P* = 0.010) as well as lower CETP activity (10.31 %, *P* = 0.009). This association remained significant in the multiple regression analysis even after adjusting for gender, age, BMI, LDL-C, triglycerides, glucose, lecithin-cholesterol acyltransferase, SBP and MetS (*P* = 0.003).

**Conclusions:**

Our data suggest a metabolic association between PCSK9 and CETP independent of lipid-lowering treatment. The clinical implications of this metabolic relationship could be relevant for explaining the effect of PCSK9 and CETP inhibition on overall lipid profiles.

**Electronic supplementary material:**

The online version of this article (doi:10.1186/s12933-016-0428-z) contains supplementary material, which is available to authorized users.

## Background

High levels of LDL cholesterol (LDL-C) and triglycerides and low levels of HDL cholesterol (HDL-C) are associated with an increased risk of cardiovascular events. Over the past few decades, mortality related to cardiovascular disease has steadily decreased in western countries; however, in recent years, the decline has been offset by the increase in obesity. Obesity is strongly associated with metabolic syndrome and the atherogenic dyslipidemia resulting from insulin resistance. While lifestyle treatments are effective, drugs targeting individual risk factors are often required. Novel approaches are directed towards the treatment of several risk factors with one drug. Despite the significant improvements many patients obtain with statins, residual risk remains for those who cannot achieve optimal levels of LDL-C or for those who are statin intolerant, underscoring the need for additional therapies to further manage this risk [1–3]. Inhibitors of proprotein convertase subtilisin/kexin 9 (PCSK9) and cholesteryl ester transfer protein (CETP) are both under current investigation as potential drugs for reducing cardiovascular disease [4, 5].

PCSK9 is mainly produced in the liver and intestine and is secreted into the circulation, where it mediates LDL receptor (LDLR) degradation in lysosomes, resulting in fewer LDLRs on the cell membrane [6]. Therefore, PCSK9 decreases LDL-C clearance from circulation and elevates plasma LDL-C. PCSK9 inhibitors increase cell surface LDLR expression and reduce LDL-C levels by more than 60 % [7]. Gain-of-function mutations in PCSK9 decrease the number of LDLRs on the cell surface and represent a rare cause of familial hypercholesterolemia [8], while loss-of-function (LOF) mutations in PCSK9 result in an increased number of LDLRs on the cell surface, leading to a lifelong low levels of LDL-C and a markedly reduced cardiovascular risk [9, 10].

CETP mediates the transport of cholesteryl ester from HDL to apolipoprotein B-containing lipoproteins such as VLDL and LDL. Animal studies, as well as clinical and epidemiologic evidence, have suggested that inhibiting CETP is an effective strategy for raising HDL-C levels and reducing LDL-C levels. Inhibiting CETP decreases the concentration of LDL-C by up to 45 % and increases the concentration of HDL-C by up to 180 % [11–14].

Recently, it has been reported that CETP inhibitors influence PCSK9 levels as an off-target effect [15–17]. Furthermore, some PCSK9 antibodies reduce the expression of SREBP-regulated genes [18]. The *PCSK9* and *CETP* genes are regulated by members of the SREBP transcription factor family [19, 20]. In vitro data have shown that CETP inhibitors decrease the occupancy of SREBP1 and SREBP2 on the sterol regulatory element within the PCSK9 promoter [21, 22]. Taken together, there is considerable interest in the relationship between CETP and PCSK9 in humans. Therefore, a treatment-independent metabolic relationship between these molecules cannot be ruled out.

Accordingly, the current study was conducted to examine the relationship between plasma CETP activity and PCSK9 levels in patients with metabolic disturbances. In addition, we studied the impact of the LOF PCSK9 genetic variant rs11591147 (R46L).

## Methods

### Subjects

We studied 450 participants (median age, 60 years; 49 % women) who attended the metabolism unit because of metabolic syndrome (MetS; 78 %) according to the ATPIII criteria, atherogenic dyslipidemia (AD; 32 %), obesity (defined as BMI ≥ 30 kg/m^2^; 50 %), type 2 diabetes mellitus (T2DM; 72 %), or other risk factors (hypercholesterolemirefa/hypertriglyceridemia; 13 %). Subjects with chronic lung, renal or liver disease; cancer; or any other serious disease were excluded. Patients on lipid-lowering drugs (N = 391) underwent a wash-out period of at least 6 weeks (8 weeks if they were on fibrates). Anamnesis and physical examination data were recorded. The Hospital Ethical Committee approved this study, and all the patients provided written consent to participate in the study.

### Blood sample collection and storage

A blood sample was obtained after overnight fasting from each patient in the study cohort. Plasma and serum aliquots were prepared and stored at −80 °C in the BioBanc at our centre until further use. Cellular buffy coats were obtained from 425 individuals, and the cells were stored at −80 °C until DNA analyses were performed.

### PCSK9, CETP activity and biochemical analysis

Biochemical parameters, lipids, and apolipoproteins were measured using colorimetric, enzymatic and immunoturbidimetric assays (Spinreact, SA, Spain; Wako Chemicals GmbH, Germany; Polymedco, NY, US; CV < 4 %) that were adapted to a Cobas Mira Plus Autoanalyser (Roche Diagnostics, Spain). The lipid profile was analysed according to Spintrol “H” CAL (Spinreact, SA, Spain) GC–MS reference methods. Spintrol “H” Normal (Spinreact, SA, Spain) was used as a quality control. All samples were subjected to the Liposcale test. This advanced lipoprotein test is based on 2D diffusion-ordered (1)H nuclear magnetic resonance (NMR) spectroscopy. This method adds diffusion coefficients to classical NMR determinations to provide a direct measure of mean particle size and number [23]. CETP activity was measured using a fluorometric assay (BioVision, CA, USA). Lecithin-cholesterol acyltransferase (LCAT) activity was assessed using a fluorometric assay (Calbiochem, CA, USA). Circulating PCSK9 levels were measured using commercial ELISA kits (R&D Systems, MN, USA).

### SNP selection and genotyping

We genotyped the rs11591147 (R46L) genetic variant of PCSK9, which was selected from the International HapMap database (http://hapmap.ncbi.nlm.nih.gov/). The LOF PCSK9 genetic variant rs11591147 (R46L) is associated with lower levels of plasma PCSK9 [24]. Genomic DNA was extracted from peripheral leukocytes that had been isolated from anticoagulated venous blood using a QIAamp DNA Blood Kit (Qiagen Iberia SL, Madrid, Spain) according to the manufacturer’s instructions. The DNA was genotyped for the genetic variant on the Sequenom MassARRAY platform using the iPLEX Gold protocol as specified by the manufacturer (Sequenom Inc., San Diego, CA) [25]. The genotypes of 5 of the samples were confirmed using duplicate Sequenom runs, and they showed 100 % consistency. Genotyping was performed at the Spanish National Genotyping Centre.

### Statistical analysis

The results are expressed as the mean ± SD for normally distributed data, the median (interquartile range) for data that was not normally distributed, and frequencies for categorical data. The differences between groups were assessed using the Mann–Whitney U test or the Kruskal–Wallis test. Correlations were evaluated using Spearman’s test. Multiple linear regression analysis was used to test the association between CETP activity and PCSK9 levels with the following covariates: age, gender, BMI, systolic blood pressure (SBP), LDL-C, triglycerides, glucose, LCAT and MetS. All the association analyses were adjusted for age, sex and BMI (when appropriate). The Hardy–Weinberg equilibrium test was performed in the studied population using a χ^2^ test. Statistical analyses were performed using SPSS software (IBM SPSS Statistics, version 22.0). All statistical tests were two-tailed and *P* < 0.05 was taken as significant.

## Results

### Participant characteristics and biochemical parameters

The clinical, metabolic and biochemical parameters of the 450 recruited subjects (male/female: 231/219) are shown in Table 1. The median age of the study participants was 60 years; 78 % had MetS, 32 % had AD, 50 % had obesity, 72 % had T2DM, and 13 % had other risk factors.

**Table 1:** Characteristics of the study subjects

Variable	N = 450
Age (years)	60 (50–66)
Gender (% men)	51.3
BMI (Kg/m^2^)	30.0 (26.9–35.0)
SBP(mmHg)	135 (125–149)
DBP (mmHg)	80 (72–86)
AD (%)	32.1
MetS (%)	78.1
Obesity (%)	50.6
T2DM (%)	72.0
Cholesterol (mmol/L)	5.20 (4.54–6.19)
Triglycerides (mmol/L)	1.54 (0.99–2.45)
LDL-C (mmol/L)	3.27 (2.55–4.07)
HDL-C (mmol/L)	1.40 (1.21–1.62)
Glucose (mg/dL)	128.5 (101.3–164.9)
Apo B100 (mg/dL)	101.0 (84.0–120.0)
Apo A1 (mg/dL)	137.9 ± 14.9
CETP activity (nmol/L per h)	11.6 (10.2–13.3)
LCAT activity (470/390 nm)	2.14 (2.07–2.24)
PCSK9 (ng/mL)	319.9 (254.4–403.9)
PCSK9 rs11591147 GG	94.8
Genotype^a^, (%) GT	5.2
Lp particle number	
VLDL (nmol/L)	49.7 (28.5–97.8)
Large VLDL (nmol/L)	1.83 (1.01–2.99)
Medium VLDL (nmol/L)	7.87 (4.52–13.6)
Small VLDL (nmol/L)	39.9 (22.7–82.2)
LDL (nmol/L)	892.3 (681.8–1175.8)
Large LDL (nmol/L)	113.4 (87.0–149.4)
Medium LDL (nmol/L)	319.4 (234.3–417.2)
Small LDL (nmol/L)	463.5 (337.9–618.5)
HDL (µL/L)	25.3 (21.6–29.4)
Large HDL (µL/L)	0.15 (0.11–0.19)
Medium HDL (µL/L)	6.94 (5.08–9.21)
Small HDL (µL/L)	17.9 (15.5–20.9)
Lp size (diameter, nm)	
VLDL	42.6 ± 0.57
LDL	21.0 (20.9–21.1)
HDL	8.19 (8.12–8.24)

The data are presented as the mean ± SD for normally distributed data, the median (IQR) for non-normally distributed data or as the percentage for categorical variables

*BMI* body mass index, *SBP* systolic blood pressure, *DBP* diastolic blood pressure, *AD* atherogenic dyslipidemia, *MetS* metabolic syndrome, *T2DM* type 2 diabetes mellitus, *LDL-C* LDL cholesterol, *HDL-C* HDL cholesterol

^a^SNP analysis was performed in 425 individuals

**Fig. 1:**
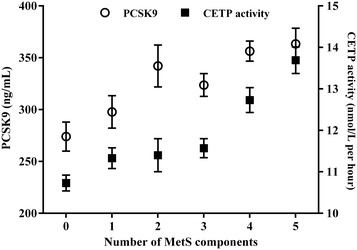
PCSK9 levels and CETP activity according to the number of MetS components (*0* 8.5 %; *1* 6.9 %; *2* 8.2 %; *3* 18.7 %; *4* 32.7 %; *5* 25.0 %). The data are presented as the mean ± SEM. *P* values were obtained using the Kruskal–Wallis test. PCSK9: *P* = 0.002; CETP: *P* < 0.0001. The univariate analysis showed that this relation persisted after adjustment for age, gender in both parameters (*P* < 0.0001)

Both PCSK9 levels and CETP activity were significantly elevated in patients with MetS [327.9 (261.9–417.9) vs 288.1 (233.7–360.9) ng/mL, *P* < 0.004, and 12.03 (10.33-14.15) vs 10.90 (10.08–12.02) nmol/L per h, *P* < 0.0001, respectively]. PCSK9 levels and CETP activity increased significantly as the number of MetS components increased (*P* = 0.002 and *P* < 0.0001, respectively) (Fig. 1). After adjustment PCSK9 levels and CETP activity for age and gender, the association with the MetS components remained significant in both parameters (*P* < 0.0001).

### Associations between CETP activity and PCSK9 levels

**Fig. 2:**
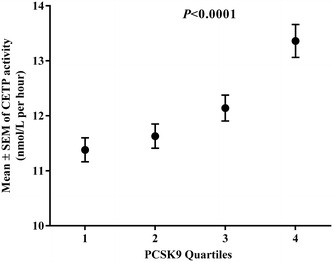
CETP activity across PCSK9 quartiles. CETP activity are presented as the mean ± SEM. *P* values were obtained using the Kruskal–Wallis test. Quartile 1 (median (IQR) (218.7 (191.2–232.6); N = 112), Quartile 2 (283.2 (269.5–299.8); N = 113), Quartile 3 (356.7 (341.2–377.8); N = 113) and Quartile 4 (471.9 (435.2–531.4; N = 112). The univariate analysis showed that this relation persisted after adjustment for age, gender, BMI, SBP, triglycerides, LDL-C and glucose (*P* = 0.003)

CETP activity stratified by quartiles of PCSK9 concentration in the whole cohort is shown in Fig. 2. A higher PCSK9 quartile corresponded with higher CETP activity (*P* < 0.001). The univariate analysis showed that this relation persisted after adjustment for age, gender, BMI, SBP, triglycerides, LDL-C and glucose (*P* = 0.003). A significant positive association between PCSK9 and CETP was observed in the whole group (r = 0.256, *P* < 0.001) (Table 2). After adjusting PCSK9 levels for age, gender, BMI, SBP, LDL-C, triglycerides and glucose, the correlation with CETP remained significant (r = 0.158, *P* = 0.003). CETP was also significantly positively associated with SBP, diastolic blood pressure, cholesterol, LDL-C, triglycerides and glucose and negatively associated with LCAT (*P* < 0.05) in the whole group. LDL particle number showed a positive association with CETP activity (*P* < 0.001), whereas there was a negative association with medium HDL particles (r = −0.193, *P* < 0.001) and positive associations with small and large HDL particles (*P* < 0.001). PCSK9 was positively associated with LDL and LDL particle number and inversely associated with LDL size. PCSK9 levels also correlated with an AD profile (Table 2).

**Table 2:** Spearman’s correlations of CETP activity and PCSK9 levels with clinical and metabolic variables

	CETP		PCSK9	
	r	*P*	r	*P*
CETP	1.000	–	0.256^a^	<0.0001
PCSK9	0.256^a^	<0.0001	1.000	–
Age	−0.088	0.063	0.045	0.346
BMI	0.020	0.679	0.052	0.268
SBP	0.208	<0.0001	0.123	0.022
DBP	0.129	0.016	0.085	0.112
Cholesterol	0.585	<0.0001	0.194	<0.0001
Triglycerides	0.491	<0.0001	0.210	<0.0001
LDL-C	0.412	<0.0001	0.179	<0.0001
HDL-C	0.134	0.004	0.109	0.021
ApoB100	0.494	<0.0001	0.194	<0.0001
ApoA1	−0.002	0.970	0.135	0.004
Glucose	0.186	<0.0001	0.215	<0.0001
LCAT	−0.251	<0.0001	−0.072	0.127
Lp particles number				
Total VLDL	0.419	<0.0001	0.176	<0.0001
Large VLDL	0.411	<0.0001	0.160	0.001
Medium VLDL	0.413	<0.0001	0.172	<0.0001
Small VLDL	0.420	<0.000	0.177	<0.0001
Total LDL	0.300	<0.0001	0.134	0.004
Large LDL	0.246	<0.0001	0.093	0.049
Medium LDL	0.234	<0.0001	0.111	0.019
Small LDL	0.332	<0.0001	0.152	0.001
Total HDL	0.098	0.038	0.024	0.614
Large HDL	0.109	0.021	0.043	0.359
Medium HDL	−0.193	<0.0001	−0.044	0.356
Small HDL	0.241	<0.0001	0.071	0.136
Lp size				
VLDL	−0.276	<0.0001	−0.114	0.015
LDL	−0.200	<0.0001	−0.107	0.023
HDL	−0.362	<0.0001	−0.104	0.027

^a^After adjusting PCSK9 for age, gender, BMI, SBP, LDL-C, triglycerides and glucose, the Spearman’s correlation with CETP remained significant (r = 0.158, *P* = 0.003)

*BMI* body mass index, *SBP* systolic blood pressure, *DBP* diastolic blood pressure, *LDL-C* LDL cholesterol, *HDL-C* HDL cholesterol

To further explore the relationship between CETP and PCSK9, a linear regression model was generated (Table 3). The significant direct relation between CETP and PCSK9 remained robust after additional adjustment for the following covariates (model 5): age, gender, BMI, SBP, LDL-C, triglycerides, glucose, LCAT and MetS (beta 0.110; *P* = 0.003).

**Table 3:** Association between CETP activity and PCSK9 levels

Adjusted for	CETP		
	Beta	*P*	*R* ^*2*^
PCSK9			
Model 1: crude (no adjustment)	0.253	<0.0001	0.064
Model 2: age, gender and BMI	0.268	<0.0001	0.085
Model 3: model 2 + LDL-C, TG, and Glu	0.107	0.001	0.582
Model 4: model 3 + SBP and LCAT	0.107	0.004	0.624
Model 5: model 4 + MetS	0.110	0.003	0.624

Linear regression analysis results are displayed as beta coefficients with the *P* and *R* ^*2*^ values for each model. CETP was the dependent variable

*BMI* body mass index, *LDL-C* LDL cholesterol, *TG* triglycerides, *Glu* glucose, *SBP* systolic blood pressure, *LCAT* lecithin-cholesterol acyltransferase, *MetS* metabolic syndrome

### Association of rs11591147 PCSK9 genetic variant with CETP activity, PCSK9 and LDL-C concentrations

**Fig. 3:**
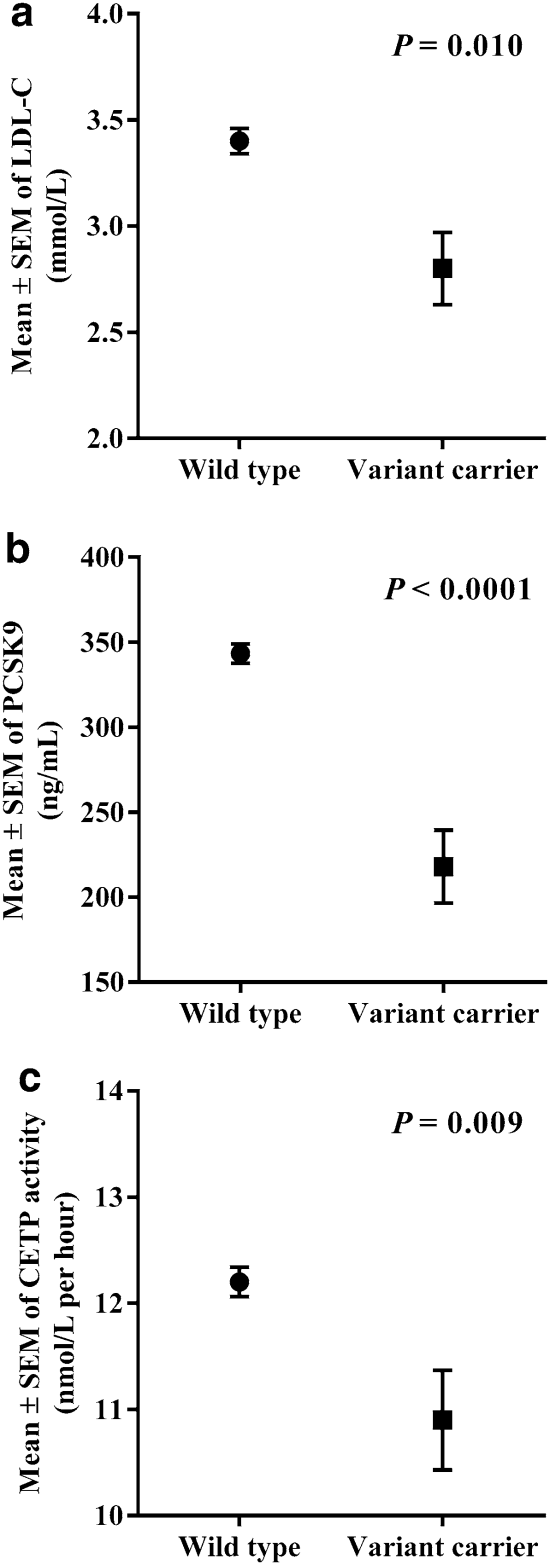
LDL-C levels (**a**), PCSK9 (**b**) levels and CETP (**c**) activity according to rs11591147 PCSK9 variant. The data are presented as the mean ± SEM. There were 403 individuals with the wild type GG genotype and 22 with the variant GT genotype. *P* values were obtained with the Mann–Whitney test

The impact of the LOF PCSK9 genetic variant rs11591147 (R46L) on CETP activity was studied in 425 patients. The frequency of PCSK9 rs11591147 (R46L) heterozygosity was 5.2 % among the whole group **(**Table 1**)**. As expected, individuals harbouring the variant GT genotype had significant lower levels of LDL-C [2.55 (2.40–3.46) mmol/L vs 3.28 (2.55–4.08), *P* = 0.010] and PCSK9 [193.6 (147.8–264.9) vs 328.8 (258.9–410.2) ng/mL, *P* < 0.0001)] than individuals with the wild type GG genotype (Fig. 3a, b, respectively). Interestingly, individuals with the variant GT genotype had significantly lower CETP activity than individuals with the wild type GG genotype [10.15 (9.59–11.93) vs 11.65 (10.31–13.50) nmol/L per h, *P* = 0.009]; this finding remained significant (*P* = 0.044) after adjusting for age, gender and BMI (Fig. 3c).

## Discussion

### PCSK9 plasma concentrations and CETP activity are directly correlated

In this study, we explored the association between circulating PCSK9 concentration and plasma CETP activity in a group of patients with increased cardiovascular risk due to metabolic alterations who were not on lipid-lowering therapies or who underwent a wash-out period. We found a moderate but significant and robust direct association between both parameters that was maintained after multiple adjustment for confounding covariates, including plasma lipid concentrations. This is the first report suggesting a direct correlation between these two biochemical parameters in humans. Moreover, our data suggest that this association is independent of intermediate factors.

Because both parameters had parallel behaviour in the presence of metabolic alterations, such as MetS components, a possible explanation could be that higher PCSK9 concentrations, which evoke higher LDL-C plasma levels, would increase CETP activity by increasing substrate concentration. Conversely, higher CETP activity would elicit higher LDL-C and PCSK9 concentrations. Abifadel et al. observed that endogenous plasma CETP activity was significantly increased in familial hypercholesterolemia patients carrying the PCSK9 Leu181Arg gain-of-function mutation. They suggest this effect could be due to an increase of plasma levels of cholesterol ester acceptors [26]. However, the association between PCSK9 and CETP activity in our study was maintained after adjusting for multiple covariates, including LDL-C plasma concentration, thereby weakening this hypothesis.

### PCSK9 and CETP share molecular regulatory mechanisms

Recently, it was reported that CETP inhibitors influence PCSK9 levels as an off-target effect. In vivo data showed that CETP inhibitors decrease PCSK9 levels in humans [17] and in rhesus macaques [16]. This effect could explain why CETP inhibitors reduce (V)LDL-C. Dong et al. demonstrated in liver cells that CETP inhibitors reduced the mature form of SREBP2, leading to attenuated transcription of LDLR and its degrader PCSK9 [22]. In addition, Miyosawa et al. demonstrated that K-312, a new CETP inhibitor, decreased the occupancy of SREBP1 and SREBP2 on the sterol regulatory element within the PCSK9 promoter [21]. Consistent with these results, van der Tuin et al. demonstrated in ApoE*-Leiden.CETP mice that anacetrapib lowers hepatic PCSK9 expression by interacting with SREBP2, leading to reduced plasma PCSK9 levels and increased hepatic LDLR increasing LDLR-mediated hepatic clearance of remnant lipoprotein and decreasing atherogenicity in animal models [15]. Although these studies suggest an effect of CETP inhibitors on PCSK9 independent of CETP itself, a metabolic association between these parameters cannot be excluded. As in the PCSK9 gene, the promoter region of the CETP gene has recognition domains for SREBP1a and SREBP2, and thus a pathophysiological parallel regulatory mechanism that affects both PCSK9 and CETP protein expression must be considered. In our study, we did not measure CETP mass; however, CETP mass and ex vivo activity were highly correlated in previous studies [27]. To confirm this finding, we analysed CETP mass and activity in a group of 30 patients with T2DM. There was a significant positive association between CETP mass and activity (r = 0.543, *P* = 0.002) in the diabetic patient group (Additional file 1: Figure S1A). In addition, PCSK9 was also significantly positively associated with CETP mass (r = 0.521, *P* = 0.003) (Additional file 1: Figure S1B), supporting the hypothesis of common gene upregulation in patients with metabolic alterations.

In our cohort, 5.2 % of the patients harboured the PCSK9 R46L LOF mutation, similar to previous data in Caucasian subjects [24, 28]. This subgroup of patients had both low PCSK9 and LDL levels, and interestingly, CETP activity was also reduced. These data support the hypothesis that the correlation between PCSK9 and CETP activity is not dependent on external environmental factors that influence these parameters. A possible mechanism could be that PCSK9 LOF mutations result in increased LDLR occupancy at the cell surface, thereby increasing LDL internalization and intracellular cholesterol concentrations and leading to lower SREBP activity, which could influence PCSK9, and possibly CETP, expression.

### PCSK9 and CETP correlation could explained therapy-induced global lipoprotein profile variations

There are several implications of this putative metabolic association that should be considered when ascertaining the impact of CETP inhibitors on PCSK9 levels that is attributed to off-target effects. PCSK9 inhibitors, in addition to their important LDL-lowering effect, induce a moderate but consistent increase in HDL cholesterol concentration. Our data could partially explain this phenomenon as a consequence of the decrease in CETP activity associated with lower PCSK9 levels.

In general, a parallel reduction of CETP activity along to lower PCSK9 levels could be seen as beneficial, however the overall effect of CETP on atherogenesis is under debate. While hypertriglyceridemia stimulates CETP enzymatic activity [29], a reduction on cholesterol ester transfer from triglyceride rich lipoprotein to HDL has been associated to coronary heart disease presence in diabetic patients [30]. If these observations are reproduced in patients on PCSK9 inhibitors treatment is not known, but it could explained the wide impact of these drugs on lipid profile affecting in a moderate way, triglycerides and HDL reinforcing the role of this treatment in patients with combined hyperlipidemias.

Our study has several limitations. It was an observational study, and thus, we cannot explain the mechanisms underlying the findings. Unfortunately, we had no access to patients on PCSK9 inhibitors who could have provided more information regarding the CETP-PCSK9 association; however, we have data on patients with genetically driven low PCSK9 levels. Our included patients with metabolic alterations, so we cannot extrapolate our findings to other populations.

## Conclusions

In conclusion, we report that PCSK9 plasma concentration is directly correlated with CETP activity in patients with metabolic imbalances. The mechanism underlying this association is not yet known, but it could explain the broad impact of drugs that regulate these two proteins on lipoprotein profiles.

## Additional file

10.1186/s12933-016-0428-z Correlations between CETP activity and CETP mass (A) and between PCSK9 and CETP mass (B) in patients with T2DM (n=30). CETP mass was measured in human serum using a quantitative enzyme-linked immunosorbent assay (ELISA) (American Diagnostica Gmbh). Spearman coefficients and *P* values are shown.

## Abbreviations

CETP: cholesteryl ester transfer protein
PCSK9: proprotein convertase subsilin/kexin 9
MetS: metabolic syndrome
LCAT: lecithin-cholesterol acyltransferase
T2DM: type 2 diabetes mellitus
AD: atherogenic dyslipidemia
LDLR: LDL receptor
BMI: body mass index
SBP: systolic blood pressure
LDL-C: LDL cholesterol
HDL-C: HDL cholesterol
LOF: loss-of-function
SREBP: sterol regulatory element-binding protein
