# Supplementary material for: Circulating PCSK9 levels and CETP plasma activity are independently associated in patients with metabolic diseases
Source: Cardiovasc Diabetol. 2016 Aug 4;15:107. doi: 10.1186/s12933-016-0428-z (PMC4973048; doi:10.1186/s12933-016-0428-z)

**Figure S1**. Correlations between CETP activity and CETP mass (A) and between PCSK9 and CETP mass (B) in patients with T2DM (n=30). CETP mass was measured in human serum using a quantitative enzyme-linked immunosorbent assay (ELISA) (American Diagnostica Gmbh). Spearman coefficients and *P* values are shown.


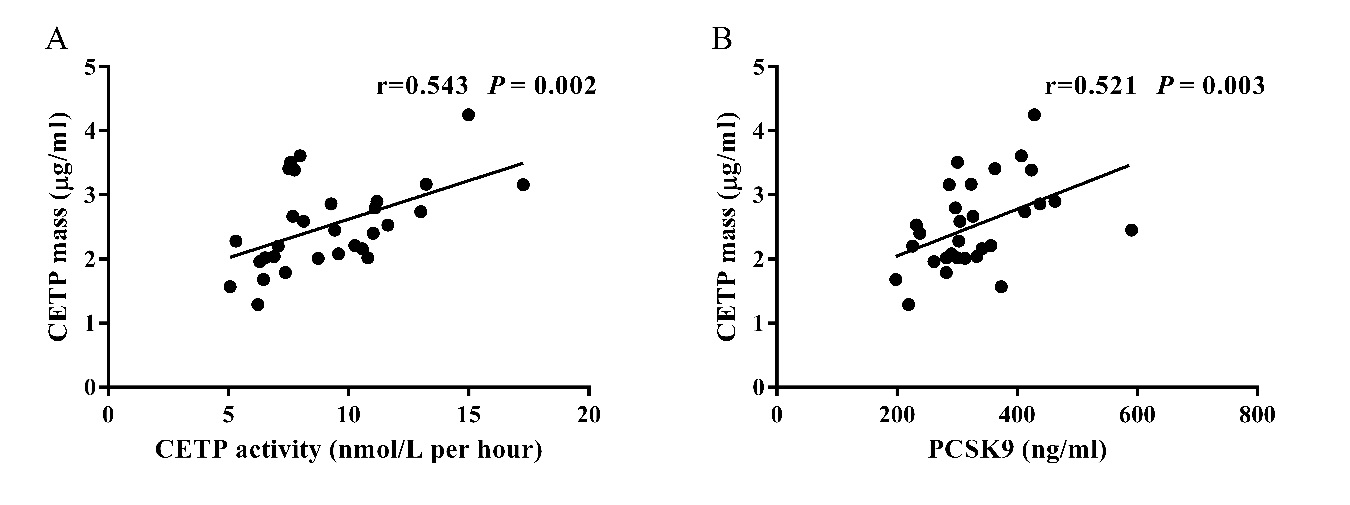

Supplement: Supplementary file 1 — 10.1186/s12933-016-0428-z Correlations between CETP activity and CETP mass (A) and between PCSK9 and CETP mass (B) in patients with T2DM (n=30). CETP mass was measured in human serum using a quantitative enzyme-linked immunosorbent assay (ELISA) (American Diagnostica Gmbh). Spearman coefficients and P values are shown. [file 12933_2016_428_MOESM1_ESM.docx]
